# Investigations on the *Tobacco Necrosis Virus* D p60 Replicase Protein

**DOI:** 10.1371/journal.pone.0080912

**Published:** 2013-11-21

**Authors:** Liang Fang, Robert H. A. Coutts

**Affiliations:** Department of Human and Environmental Sciences, University of Hertfordshire, Hatfield, Hertfordshire, United Kingdom; University of Kansas Medical Center, United States of America

## Abstract

*Tobacco Necrosis Virus* D (TNV-D), in the genus *Betanecrovirus* (family *Tombusviridae*), possesses a single-stranded, positive-sense RNA genome containing six open reading frames (ORFs). Two 5'-proximal ORFs (1 and 2) encode overlapping polypeptides of 22 and 82 kDa (p22 and p82, respectively) which are both required for replication. The p22 auxiliary protein contains no replication motifs but the C-terminal region, downstream of a leaky stop codon, encodes a 60 kDa polypeptide (p60) which contains conserved RNA-dependent RNA polymerase (RdRP) motifs. Here we have expressed and purified recombinant p60 and show that *in vitro* it binds and efficiently synthesises both TNV-D RNA and *Satellite* tobacco necrosis virus C RNA. Alanine scanning mutagenesis of conserved amino acids in characteristic motifs in p60 revealed that some mutations significantly reduced RNA synthesis but mutating the second asparagine residue in the conserved GDD box was lethal. The effects of mutating identical amino acids in p60 on virus replication *in vivo* were examined in *Nicotiana benthamiana* plants following infection with RNA transcribed from wild type (wt) and mutant constructs. In inoculated leaves the behaviour of the mutants paralleled the *in vitro* data but systemic infection was precluded in all but one mutant which had reverted to wt. This study is the first to demonstrate the nucleic acid-binding and synthetic capabilities of a betanecrovirus polymerase.

## Introduction

**Figure 1:**
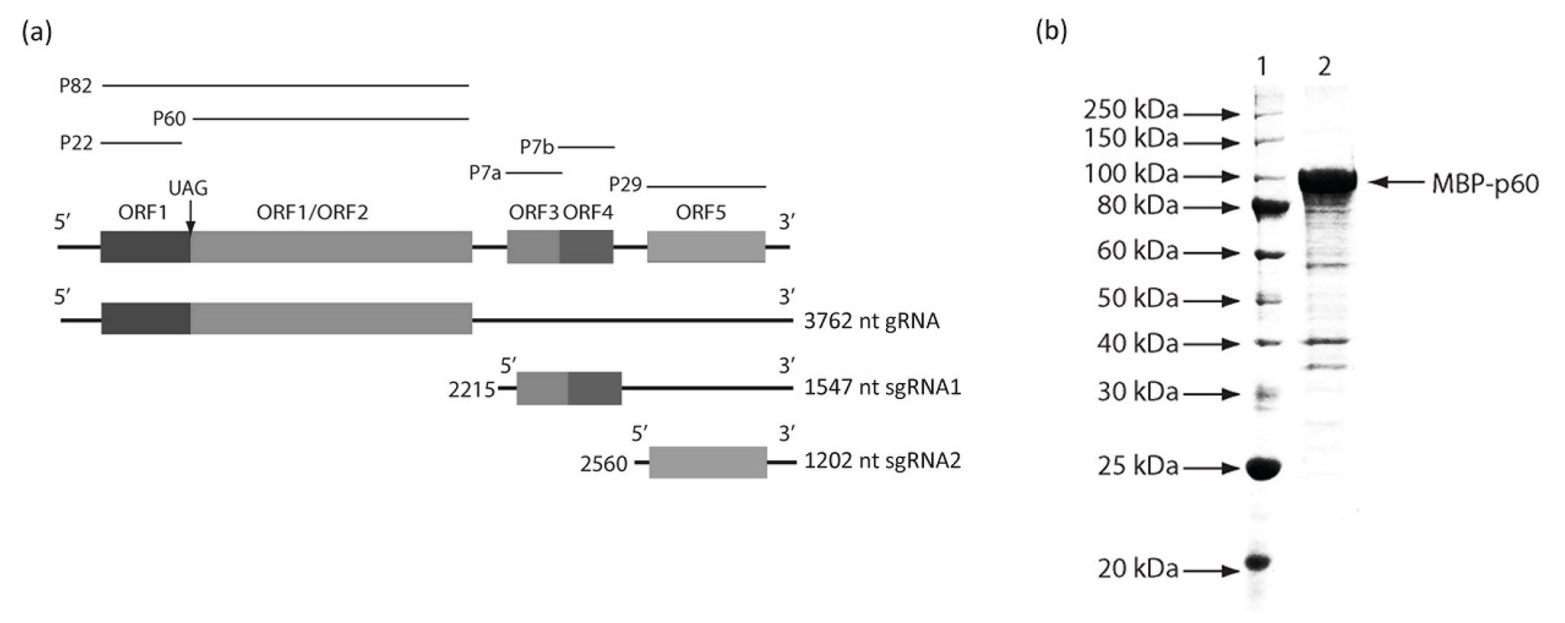
Schematic representation of TNV-D genomic and sub-genomic RNAs and expression and purification of recombinant MBP-TNV-D p60. In panel (a) open boxes correspond to predicted ORFs including ORFs 1 and 2 which encode proteins p22 and p82 respectively. Protein p60 is predicted from the sequence downstream of the leaky UAG amber stop codon [3]. The genomic and sub-genomic TNV-D RNAs encoding the virus proteins and their sizes are shown ABOVE the gene map. Panel B shows expression and purification of recombinant MBP-TNV-D p60 from *E. coli*. The MBP-p60 fusion protein was expressed at 25°C for 4 h following induction with 0.5 mM IPTG. Proteins were analysed by 10% SDS-PAGE and subjected to Coomassie blue staining. Lane 1 contains protein markers with their molecular masses (in kDa) indicated on the left. Lane 2 contains highly purified MBP-p60 following Superose 6 10/300 column purification.

Replication of RNA plant viruses is performed by membrane-bound complexes consisting of virus- and host-encoded proteins [1] where viral RNA genome multiplication requires specific interaction between the viral RNA-dependent RNA polymerase (RdRP) and its cognate RNA. Tobacco necrosis virus D (TNV-D) is a member of the genus *Betanecrovirus* in the family *Tombusviridae* [2] and possesses a monopartite genome composed of a single-stranded (ss), positive-sense genomic RNA that harbours six open reading frames (ORFs) [3,4], (Figure 1A) The translation products of the two 5'-proximal ORFs, ORFs 1 and 2, are proteins of 22 and 82 kDa (p22 and p82), respectively and both are indispensable for viral replication [5,6]. By analogy with the closely related betanecrovirus, Beet black scorch virus (BBSV) [7], and TNV- D^H^ [6], three internal ORFs encode small proteins that are required for viral cell-to-cell movement and the 3'-proximal ORF encodes the virus capsid protein (CP) which appears to be located in the cell nucleus [8]. The cell-to-cell movement proteins and the CP are translated from two different subgenomic RNA (sgRNA) elements, designated sgRNA1 and sgRNA2, respectively [4] (Figure 1A). As occurs in most members of the family *Tombusviridae*, TNV-D ORF 2 must be translated from the genomic RNA by readthrough of the leaky stop codon of ORF 1 and, thus, p82 contains the sequence of p22 at the N-terminus while its C-terminal portion contains the signature motifs of RdRPs [9,10]. These motif sequences are present in many polymerases in the ‘palm subdomain’ which is composed of a four-stranded antiparallel beta-sheet with two alpha-helices and includes the motifs A, B and C. Motif A (D-X (4,5)-D) and motif C (GDD) are spatially juxtaposed and the aspartic acid residues of these motifs are implicated in binding Mg^2+^ and/or Mn^2+^. The asparagine residue of motif B is involved in selection of ribonucleoside triphosphates over dNTPs together with the D motif. The organisation of the domain and the 3D structure of the catalytic centre of a wide range of RdRPs, even those with a low overall sequence homology, are conserved. The catalytic centre is formed by several motifs containing a number of conserved amino acid residues. The E motif is unique to RdRPs and reverse transcriptases. Based on the presence of these motifs, the predicted function of p82 is to synthesise viral RNA progeny and subgenomic RNAs for the expression of the internal and 3'-terminal genes. Studies on RdRP function in tombusviruses have included investigations on *Turnip crinkle virus* [11], Tomato bushy stunt virus (TBSV), [12], [13], *Cucumber necrosis virus* [14] and *Panicum mosaic virus* [15] but these investigations have largely focussed on the N-terminal auxiliary proteins, which appear to be the master regulators of tombusvirus replication involved in selective binding to the tombusvirus (+) RNA and recruitment of the viral RNA into replication [16,17], with less emphasis on the readthrough C-terminal regions which contain the RNA polymerase motifs. To investigate and dissect the functions of the RdRP proteins in detail most investigations used recombinant RdRPs expressed and purified from *Escherichia coli*. We have now used this approach to investigate for the first time the RdRP from the betanecrovirus, TNV-D concentrating on the C-terminal region of ORF 2, and also studied the effects of mutations in this domain on both *in vitro* synthetic capability and *in vivo* replication. 

## Materials and Methods

### Recombinant plasmids

Plasmids pTNVD and pSTNVC, which respectively contain wild type (wt) full-length TNV-D and Satellite tobacco necrosis virus C (STNV-C) cDNAs, inserted into a pUC19 derivative downstream from a T7 RNA polymerase promoter, have been described previously [18]. Plasmid pTNVD was used as a template for PCR with oligonucleotide primers TNVD-1 and TNVD-2 (Figure S1) which respectively introduced unique *Nco*I and *Hind*III sites bordering the sequence of the wt p60 region of the TNV-D RdRP p82 ORF. Reaction mixtures contained 50-200 ng pTNVD, 1 × *Pfu* buffer containing 2 mM MgSO_4_ (Promega), 200 pmol of each oligonucleotide primer, 400 µM of dNTPs (Promega), 3 U of *Pfu* polymerase (Promega) in a total volume adjusted to 50 µL with water. Reactions were conducted with initial denaturation at 95°C for 2 min then 32 cycles of 95°C for 30 sec, 58°C for 30 sec, and 72°C for 1 min/kb with a final cycle of 72°C for 10 min. PCR amplification was performed and amplicons were purified and cloned into the *E. coli* pMBP-Parallel 1 vector [19] to give pMBP-p60 for the expression of wt TNV-D p60. Also mutations were introduced into MBP-p60 to change the codons of seven individual, conserved amino acids into those specifying alanine using a *Dpn*I mediated mutagenesis PCR protocol [20] with appropriate primers listed in Figure S2. PCR was performed in the same reaction mixture and volume as above with substitutions of 5-20 ng of pMBP-p60, 200 µM of dNTPs and 250 pmol of each oligonucleotide primer and reactions were carried out following initial denaturation at 95°C to 2 min and then 18 cycles of 95°C for 30 sec (to prevent clonal expansion of any undesired second-site mutations), 55°C for 1 min, and 68°C elongation for 2 min/kb of plasmid length. Purified amplicons were cloned into pMBP-Parallel 1 to give pMBP-p60-P165A; -R166A; -R230A; -N299A; -D327A; -F370A and -R386A which were numbered and named according to the mutated amino acid position in p60 and used for the expression of mutant p60 proteins. The same mutations to p60 as described above were introduced into pTNVD to produce recombinant plasmids using the oligonucleotide primers listed in Figure S2. These plasmids were used for transcription of mutant RNA and comparison with wt TNV-D RNA in inoculated plants. All constructs was verified by DNA sequencing with an ABI PRISM DNA sequencer 377 (Perkin-Elmer). 

### Purification of p60 and derivative proteins from *Escherichia coli*

The pMBP-Parallel 1 based recombinant plasmids were used to transform *E. coli* BL21-CodonPlus (DE3)-RIPL cells (Agilent Technologies). The maltose-binding protein (MBP)-p60 fusion protein and mutant derivatives, whose expression was induced in the transformed bacteria with isopropyl beta-D-thiogalactopyranoside (IPTG) at 0.5 mM for 4 h at 25°C, were affinity purified by FPLC using MBPTrap HP columns (GE Healthcare). Recombinant proteins were concentrated by centrifugation using Amicon® Ultra-4 Centrifugal Filter Units (Millipore) containing a 10 kDa cut-off membrane and purified further by HPLC using Superose 6 10/300 columns (GE Healthcare). All operations were carried out according to the manufacturer's instructions. Recombinant protein concentrations were quantified using the Edelhoch formula [21] and analysed by 10% denaturing SDS-poly acrylamide gel electrophoresis (PAGE) after Coomassie brilliant blue staining (e.g. Figure 1B). 

### Preparation of labelled and unlabelled nucleic acids

Both labelled and unlabelled ssRNAs for RdRP assays, electrophoretic mobility shift assay (EMSA), competition experiments and plant inoculation were generated by *in vitro* transcription with T7 RNA polymerase using the HiScribe T7 *in vitro* transcription kit according to the manufacturer's recommendations (NEB). Negative (-) -strand RNA transcripts were synthesised from PCR-derived amplicons generated from respectively pTNVD and pSTNVC using oligonucleotide primer pairs TNVD-3 and TNVD-4 for TNV-D (virD (-)) and STNVC-1 and STNVC-2 for STNV-C satC (-) (Figure S1) which placed the T7 promoter at the 5’-terminus of both full-length cDNAs. To synthesise labelled transcripts 3.2 nmol [32P] UTP (3000 Ci/mmol^-1^; PerkinElmer) was included in the transcription reactions. In all cases, template DNA was eliminated by RNase-free DNase I treatment and the transcripts were extracted using phenol/chloroform and filtered through a Sephadex G-50 spin column to remove free nucleotides. The amounts and integrity of RNA transcripts were determined following 5% PAGE. 

### RNA binding studies

For EMSA, [32P]-labelled ss satC- RNA probe (28.4 pmol) was mixed with different amounts of MBP-p60 protein (0-120 pmol=0-13 μg) in 20 µL binding buffer (50 mM Tris/HCl, pH 8.2, 10 mM MgCl_2_, 1 mM EDTA, 10% glycerol and 2 U RNase inhibitor (Promega) and incubated at 25°C for 45 min. In competition experiments, different amounts of unlabelled nucleic acids were added simultaneously with the labelled ssRNA probe to the binding mixtures. In competition experiments, the same amounts (50 nM) of template RNA and increasing amounts (between 1- and 10-fold excess) of competitor RNAs were mixed. After the binding reactions, samples were analysed by non-denaturing PAGE (4.5%) in TG buffer (25 mM Tris/HCl, 192 mM glycine, pH 8.3). Gels were vacuum-dried and radio-labelled free and bound RNAs were detected and analysed by phosphorimaging using the AIDA image analyser version 3.52 software package. 

### RdRP assays

RdRP reactions were carried out as previously described [11] in the presence of 50 mM Tris-HCl (pH 8.2), 10 mM MgCl_2_, 10 mM dithiothreitol, 100 mM potassium glutamate, 1.0 mM each ATP, CTP, and GTP, 0.01 mM UTP (final concentration), and 0.5 µ of [^32^P] UTP, 30 pmol template RNA and MBP-p60 protein in a 50 µL total volume. After phenol-chloroform extraction and ammonium acetate-isopropanol precipitation, half the amount of the RdRP products was treated with S1 nuclease as described before [22]. Subsequently, the RdRP products were mixed with gel loading buffer II (Ambion) and separated on denaturing 5% polyacrylamide-8 M urea gels, followed by phosphorimager analysis.

### Inoculation of *Nicotiana benthamiana* plants, analysis of viral infection and isolation of revertants

Transcripts synthesised *in vitro* from wt clone pTNVD and the seven mutant constructs were synthesised as described above and mechanically inoculated onto *N. benthamiana* plants (three leaves per plant employing approximately 10 μg of transcript per leaf) as described before [18]. Plants were then maintained under greenhouse conditions (16 h days at 24°C, 8 h nights at 20°C) and monitored for symptom appearance from 7 to 15 days post inoculation. Total RNA preparations from *N. benthamiana* leaves were isolated using the TRI Reagent according the manufacturer’s instructions (Sigma) and northern blot analysis was performed with oligolabelled [^32^P]-radioactive DNA probes derived from the complete TNV-D genome as described previously [18]. Any mutant revertants were identified by RT-PCR using RNA isolated from both inoculated and newly developed leaves as template for the synthesis of cDNA clones corresponding to an 848 bp amplicon sequence within the TNV-D RdRP ORF (nt 1039-1887) which encompassed all of the mutated amino acids. Amplicons were cloned into pGEM®-T Easy (Promega) and sequenced as above.

## Results

### Expression and purification of MBP-TNV-D p60 in *Escherichia coli*

**Figure 2:**
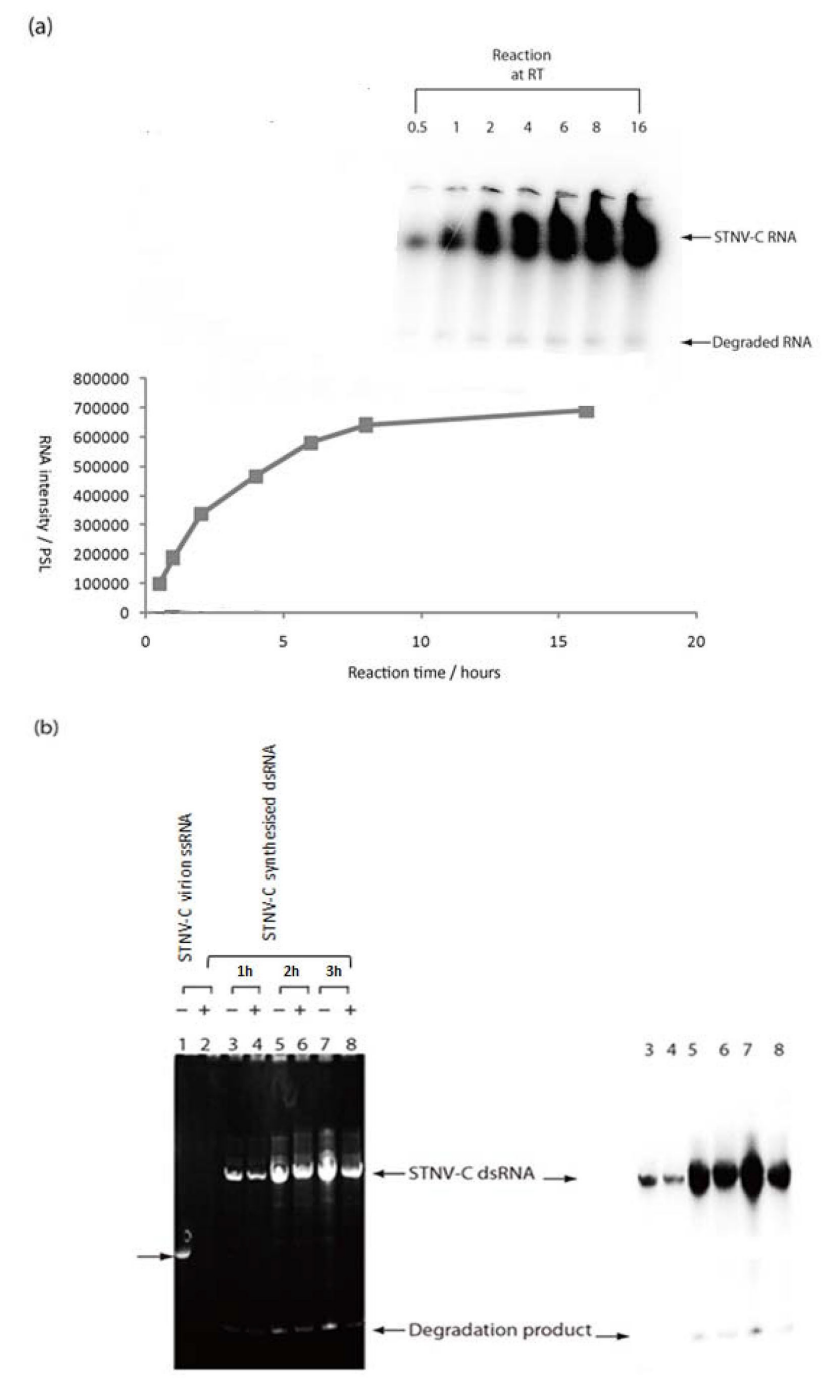
Kinetics of RdRP activity of MBP-TNV-D p60 and effects of S1 nuclease treatment on radiolabelled RNA products. Panel (a) shows representative denaturing gel analysis of radiolabelled RNA products synthesised at 25°C (RT) by *in* *vitro* transcription using satC- RNA as the template over a 16 h time course. The data is also shown in graphical form quantified by AIDA following phosphorimager analysis. Panel (b) shows the effects of S1 nuclease treatment on radiolabelled RNA products synthesised at 25°C. Products synthesised following 1, 2 or 3 h incubation were untreated (lanes 3, 5 and 7 respectively) or treated with S1 nuclease (lanes 4, 6 and 8 respectively). Radiolabelled products were stained with ethidium bromide in the left hand panel which also shows the template satC- RNA used in these reactions, untreated (lane 1) or treated (lane 2) with S1 nuclease, which is arrowed. Phosphorimaging of the gel is shown in the right hand panel.

The N-terminal region of TNV-D p82 ORF 1 encodes the p22 protein which is an auxiliary protein and the C-terminal ORF encodes the p60 protein which is the polymerase domain of p82 (Figure 1A). TNV-D p60 was successfully expressed as a C-terminal fusion protein with the MBP in *E. coli* as described in the Materials and Methods. After affinity-based purification of the MBP-p60 fusion protein (Figure 1B) its RdRP activity was tested using virD- or satC- RNA which from previous studies were shown to be the best templates for transcription. The RdRP products were analysed by PAGE, which resulted in the production of two major bands for each template. Under optimal conditions (established with satC- RNA as template) reactions were performed at 25°C with 5 µg of MBP-p60 fusion protein which over a time course reached a plateau after 6 to 8 h incubation (Figure 2A). S1 nuclease digestion of the RdRP products revealed that the major product was virtually nuclease-resistant, template-sized cRNA whilst a smaller product may be a degradation product or the result of ‘’aberrant initiation’’ by the RdRP or that the T7 transcript contains other than full-length products, which occurs frequently (Figure 2B). 

### *In vitro* RNA binding by recombinant p60

**Figure 3:**
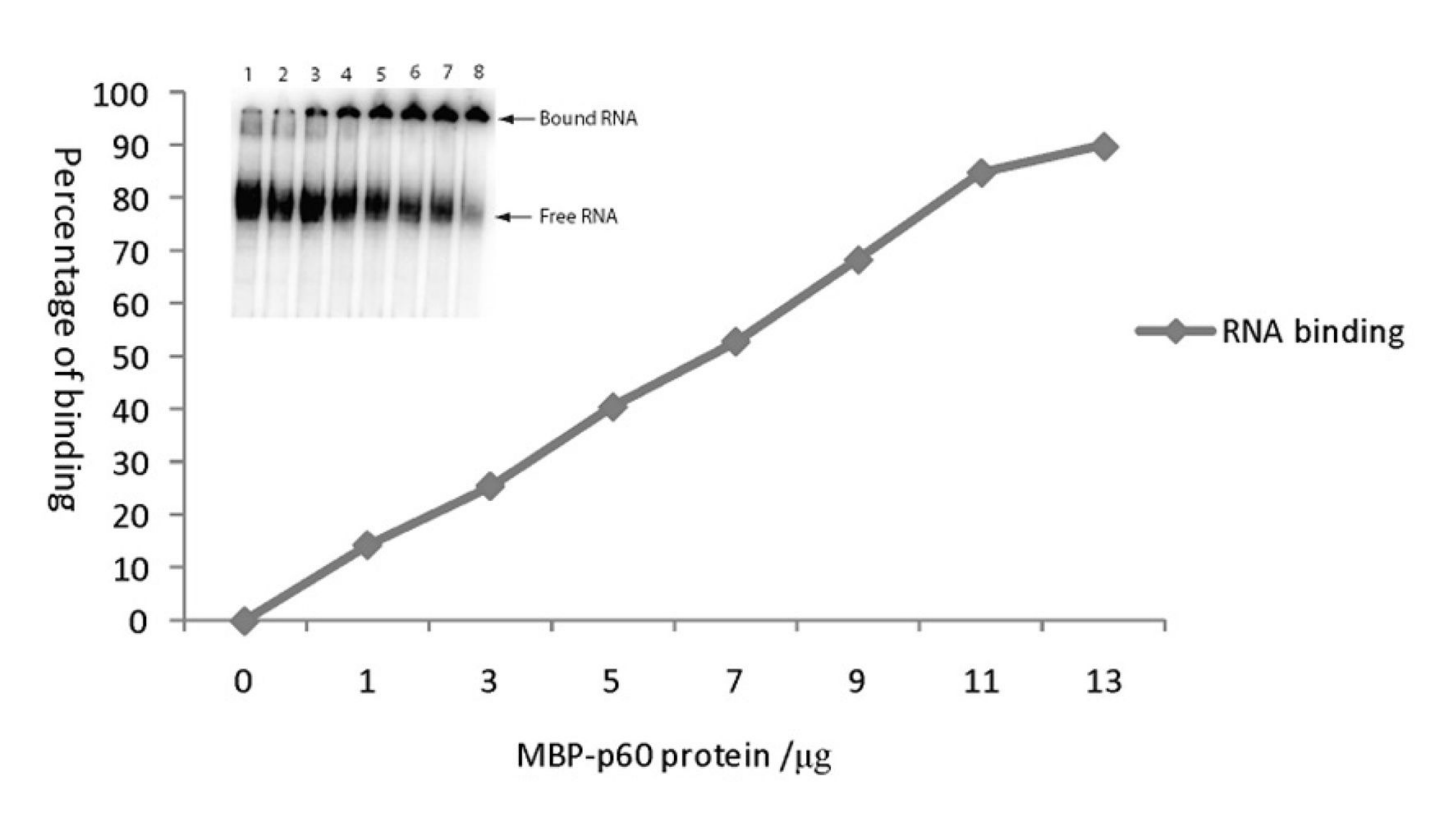
Analysis of RNA-binding properties of MBP-TNV-D p60 *in* *vitro*. Representative electrophoretic mobility shift assay (EMSA) showing interactions between recombinant p60 and ss RNA. The [32P]-labelled satC- RNA probe (28.4 pmol) was incubated with no protein (lane 1) or with MBP-p60 fusion protein at 1, 3, 5, 7, 9, 11 or 13 μg concentration (lanes 2-8, respectively). The unbound (free) RNA probe and the shifted (bound) RNA complexes are marked on the right. The data is expressed in graphical form following phosphorimager analysis and quantification by AIDA.

The potential nucleic acid-binding capability of p60 was investigated by EMSA using a [32P]-labelled ss satC- RNA probe and increasing amounts of recombinant MBP-p60 fusion protein. This approach showed that p60 could bind RNA efficiently *in vitro*. The protein retarded the probe at a concentration of 9.2 pM and retarded it completely at and above a concentration of 120 pM (Figure 3). The RNA bound to p60 did not move from the well, probably due to the large size of the complex. When the MBP-p60 fusion protein was replaced by MBP in control gel shift assays, no retardation of the probe was observed (results not shown) indicating that the association between p60 and the ss RNA was not an artefact of non-specific protein–RNA interactions. No intermediate shifted bands (resulting from limited binding of the probe by p60) were detected in the EMSA, consistent with cooperative binding of p60, where most of the RNA is either covered by p60 or not bound to it at all. All experiments were repeated three times with identical results. 

RNA binding by the MBP-p60 fusion protein was also tested in competition assays in which increasing amounts of unlabelled competitor RNAs, including positive-strand STNV-C (satC+) RNA produced from pSTNVC following linearisation with *Xho*I, virD- RNA or TBSV negative-strand RNA, were used in addition to a constant amount of labelled satC- RNA. These experiments demonstrated that satC+ and virD- RNAs were efficient competitors but that TBSV was a poor competitor (results not shown). 

### Characterisation of RdRP activities of wild type and mutated TNV-D p60

**Figure 4:**
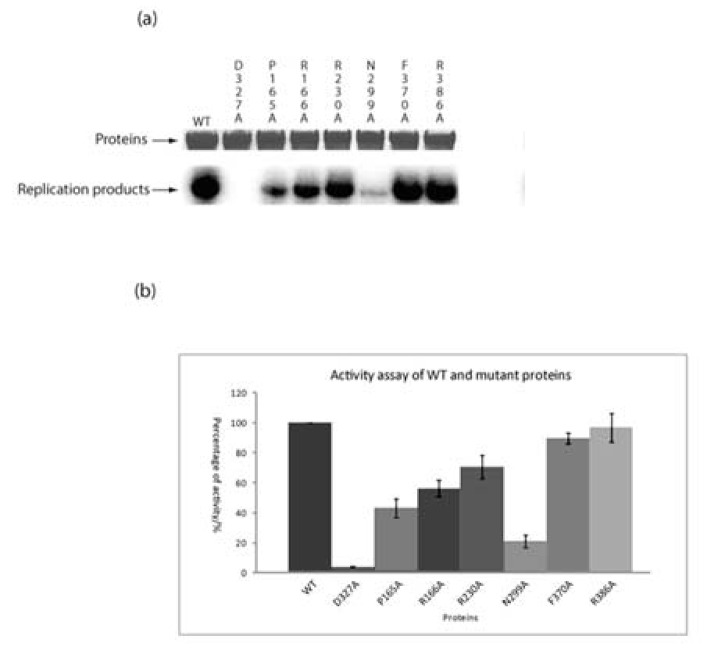
Effect of mutations to MBP-p60 on the synthesis of STNV-C RNA. The upper tier in panel (a) shows 10% SDS-PAGE analysis of the expressed proteins, the lower tier shows 5% denaturing polyacrylamide-8 M urea gel analysis of radio-labelled STNV-C dsRNA. Individual mutations are shown above the respective lanes. Radioactivity was quantified by AIDA and expressed as a percentage of the radio-labelled dsRNA produced by wt MBP-p60 in four parallel assays and the data presented as histograms in panel (b).

The RdRP activity of seven alanine mutants of amino acids present in conserved motifs found in the p60 polymerase domains of TNV-D, two other betanecroviruses, *Leek white stripe virus*, BBSV and TBSV (Figure S3) were compared to the wt enzyme using satC- RNA as template. All experiments were conducted in quadruplicate. The results showed that disruption of several conserved amino acids had significant effects on RdRP activity (Figure 4, panels A and B). The D327A mutant had no RdRP activity whilst the activity of the N299A mutant was only 20% of the wt enzyme. Three other mutants (P165A, R166A and R230A) also had significantly reduced activities while the remaining F370A and R386A mutants were reduced in activity but not significantly as compared to wt. The results using virD- RNA as template were almost identical (results not shown).

### Infectivity and symptoms of wild type TNV-D and TNV-D RdRP mutants in *Nicotiana benthamiana* plants and recovery of revertants 

**Figure 5:**
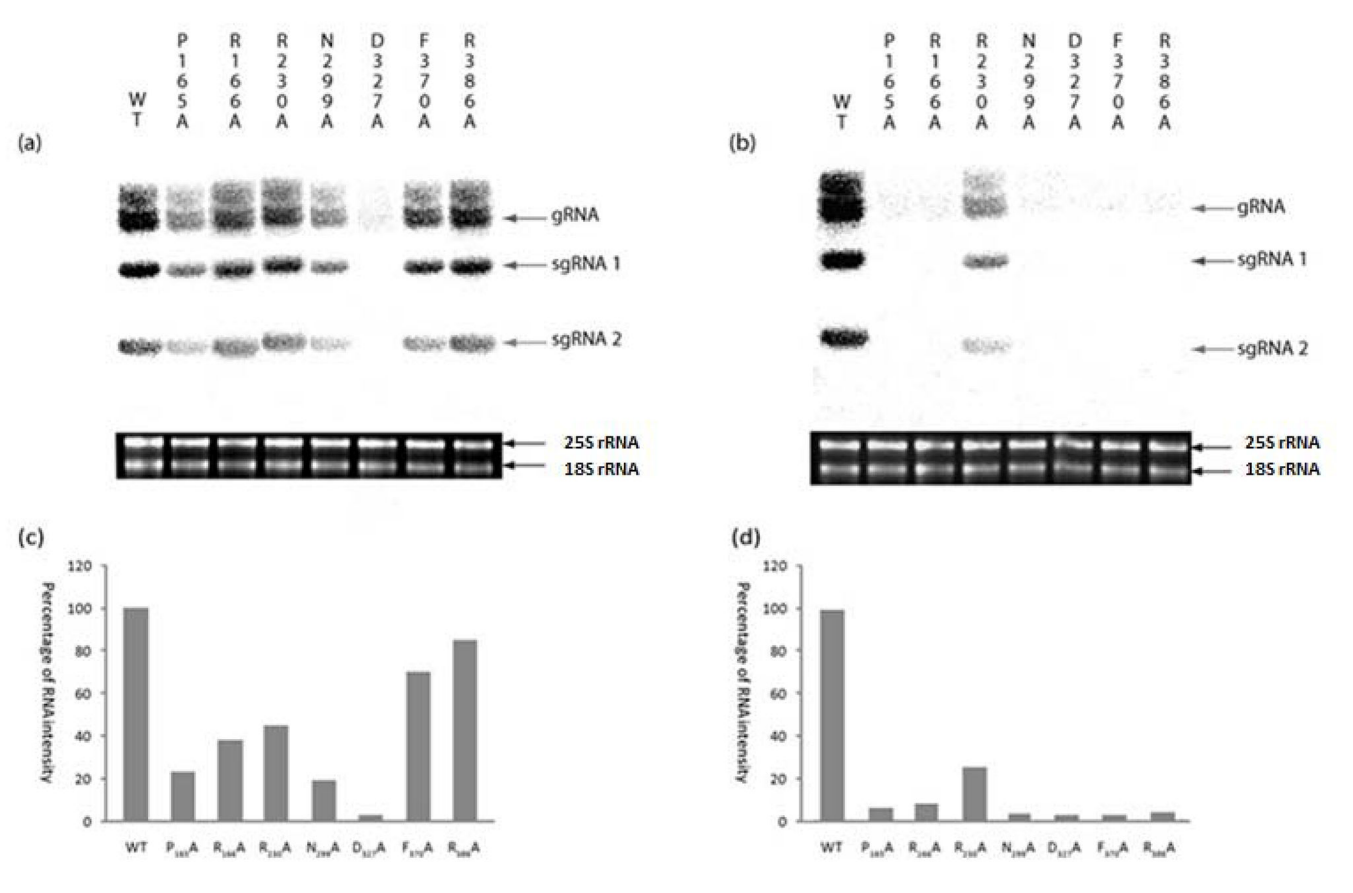
Effect of mutations in the TNV-D p60 domain on RNA replication in *Nicotiana benthamiana*. Total RNA extracted from inoculated leaves in panel (a) and systemically infected leaves in panel (b) following inoculation with wt- and mutant RNA transcripts was fractionated by agarose gel electrophoresis and analysed by northern blot hybridisation. TNV-D genomic RNA and two sub-genomic RNAs are indicated by arrows. The tobacco 25S and 18S rRNAs were stained with ethidium bromide for loading purposes. Radioactivity was quantified by AIDA and the results with inoculated and systemically infected leaves are presented as histograms in panels (c) and (d) respectively.

Identical alanine mutations to those described above for *in vitro* synthesis of RNA were introduced into a full-length, infectious clone of TNV-D and the infectivity of wt and mutant, run-off RNA transcripts compared in *N. benthamiana* plants. All of the mutants had the effect of lowering the synthesis of genomic and sub-genomic TNV-D RNAs in inoculated leaves (Figure 5, panels A and C). The D327A mutant was replication incompetent and the P165A and N299A mutant infectivity was *ca.* 20% of wt TNV-D RNA. The infectivity of mutants R166A and R230A was *ca.* 40% of wt TNV-D RNA and the infectivity of the remaining F370A and R386A mutants was *ca.* 80% of wt TNV-D RNA. The ability of all of the mutants to cause systemic infection in the inoculated plants was compromised and only the RNA of mutant R230A could be isolated in measurable amounts from the first developed, systemically infected leaves (Figure 5, panels B and D). These experiments were duplicated with essentially identical results. Putative mutant revertants were identified following cDNA cloning of the RNA isolated from all inoculated and systemically infected leaves. Only one mutant reverted to the wt TNV-D sequence where reversion of alanine to the original arginine was found in 1/3 and 2/3 clones produced from respectively inoculated and systemically infected leaves inoculated with the R230A mutant. 

## Discussion

Following the successful expression and purification of a recombinant TNV-D p60 enzyme, which binds and replicates both TNV-D and STNV-C RNA, we wished to understand more of the involvement of the RdRP A-F motif sequence motifs [10,12] in the synthetic activity of the TNV-D RdRP p60 domain. To achieve this, a series of mutant recombinant proteins were expressed and assayed together with identical mutations for the *in vitro* transcription of infectious RNA for plant bioassay. Similar investigations on TBSV RdRP concerned RNA binding by recombinant RdRPs [12] and mutations to the highly conserved GDD motif and their effects on infectivity [13]. Interestingly protein stability problems during expression in *E. coli* precluded extensive studies with mutant versions of the non-overlapping C-terminal domain of TBSV p92 (p92C) [13], which were not encountered in our study with TNV-D and the pMBP vectors.

As anticipated the TNV-D C domain mutation D327A was lethal for both RNA synthesis and plant infectivity (Figures 4 and 5 respectively). Mutations to the glycine residue in the C-domain located GDD box of tombusviruses appear to be flexible and revert to wt [12] however there is a strict requirement for the first aspartate residue, which is involved in the catalytic activity and metal ion coordination of all RdRPs [10].

Alignment of the polymerase domains of TNV-D, two other betanecroviruses, *Leek white stripe virus*, BBSV and TBSV (Figure S3) revealed that the N-terminal region, the F domain, contains a large number of positive charged residues. In TBSV both this region, known as RBR2, and p92C, which flank the conserved RdRP motifs on both sides, bind RNA and may also be involved in rNTP recruitment [12]. Two TNV-D F domain mutants pMBP-p60-P165A and -R166A had significantly reduced RdRP synthetic activity and infectivity for plants as compared to the wt. However neither mutation was lethal in TNV-D (Figures 4 and 5) suggesting that co-operation between other amino acids rather than those mutated maintains reduced synthetic capability and infectivity as has been suggested for TBSV [12]. 

Indeed similar conclusions can be drawn for all the other TNV-D mutants we investigated. For instance it would appear that other amino acids present in motifs A and B act with varying degrees of efficiency to compensate for respectively the R230A and N299A mutations where the latter caused the most dramatic effects on RNA synthesis and infectivity (Figures 4 and 5) but neither were lethal. Interestingly mutant R230A, which had reduced RNA synthetic activity (Figure 4), was the only mutant to systemically infect *N. benthamiana* (Figure 5) presumably because it had reverted to wt.

Motifs A, B, C, and D located in the catalytic portion of the palm domain of all RdRPs interact with motif E *via* hydrophobic bonds and the two E motif mutants we tested, F370A and R386A respectively were not significantly impaired in RNA synthetic activity (Figure 4) and caused minor negative effects on infectivity (Figure 5), confirming the flexibility of other residues in compensating for these mutations. Having previously demonstrated the synthetic capability of plant-derived TNV-D RdRP [23] we have now successfully expressed and purified an active recombinant form of the enzyme which will assist in further dissection of the various components of the TNV-D replicase. 

## Supporting Information

Figure S1 **Primers used for recombinant plasmid construction, protein expression and for the generation of (-** **) strand RNA templates**.
(TIF)

Figure S2 **Primers used for alanine scanning site-directed mutagenesis of TNV-D p60.**
(TIF)

Figure S3 **Amino acid alignment of the putative polymerase domains from members of the genus *Betanecrovirus* and TBSV which are most closely related to TNV-D.** Accession numbers of each virus are shown in brackets: Leek white stripe virus (LWSV; NC001822) TNV-D (D00942), Beet black scorch virus (BBSV; FN565520) and TBSV (M21958). The amino acid sequences depicted show the readthrough domain of the putative RdRPs immediately downstream of the amber termination codon and extend to the termination codon of the ORFs. The sequences were aligned using the Fast Fourier Transform MAFFT program L9INS-1 [24]. The conserved motifs (A-E) within the domains [10] are underlined in the TNV-D sequence and the amino acids mutated in this investigation are boxed. The RNA binding motif, RBR2 (F motif) is boxed in the TBSV sequence and the RdRP palm sub-domains [13] are underlined. Asterisks signify identical residues; colons signify highly conserved amino acid residues; single dots less conserved, but related residues in the four sequences.
(TIF)
