# Supplementary figures and images for: Investigations on the Tobacco Necrosis Virus D p60 Replicase Protein
Source: PLoS One. 2013 Nov 21;8(11):e80912. doi: 10.1371/journal.pone.0080912 (PMC3836746; doi:10.1371/journal.pone.0080912)

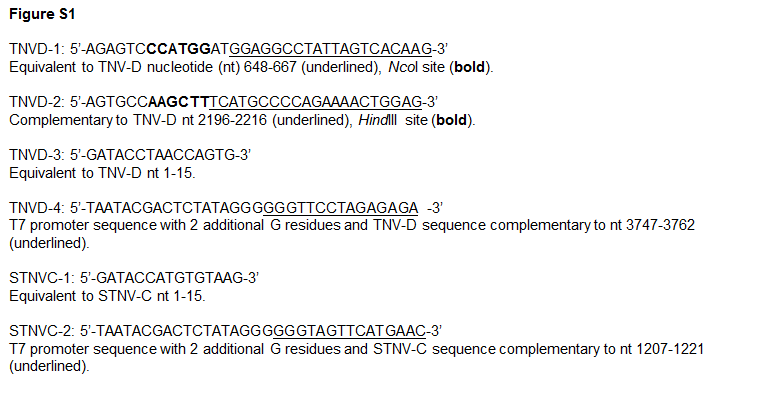

Supplement: Figure S1 — Primers used for recombinant plasmid construction, protein expression and for the generation of (- ) strand RNA templates. (TIF) [file pone.0080912.s001.tif]

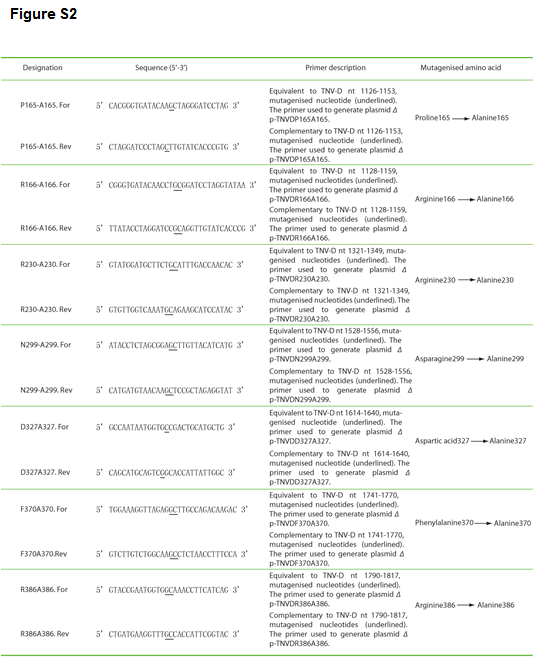

Supplement: Figure S2 — Primers used for alanine scanning site-directed mutagenesis of TNV-D p60. (TIF) [file pone.0080912.s002.tif]

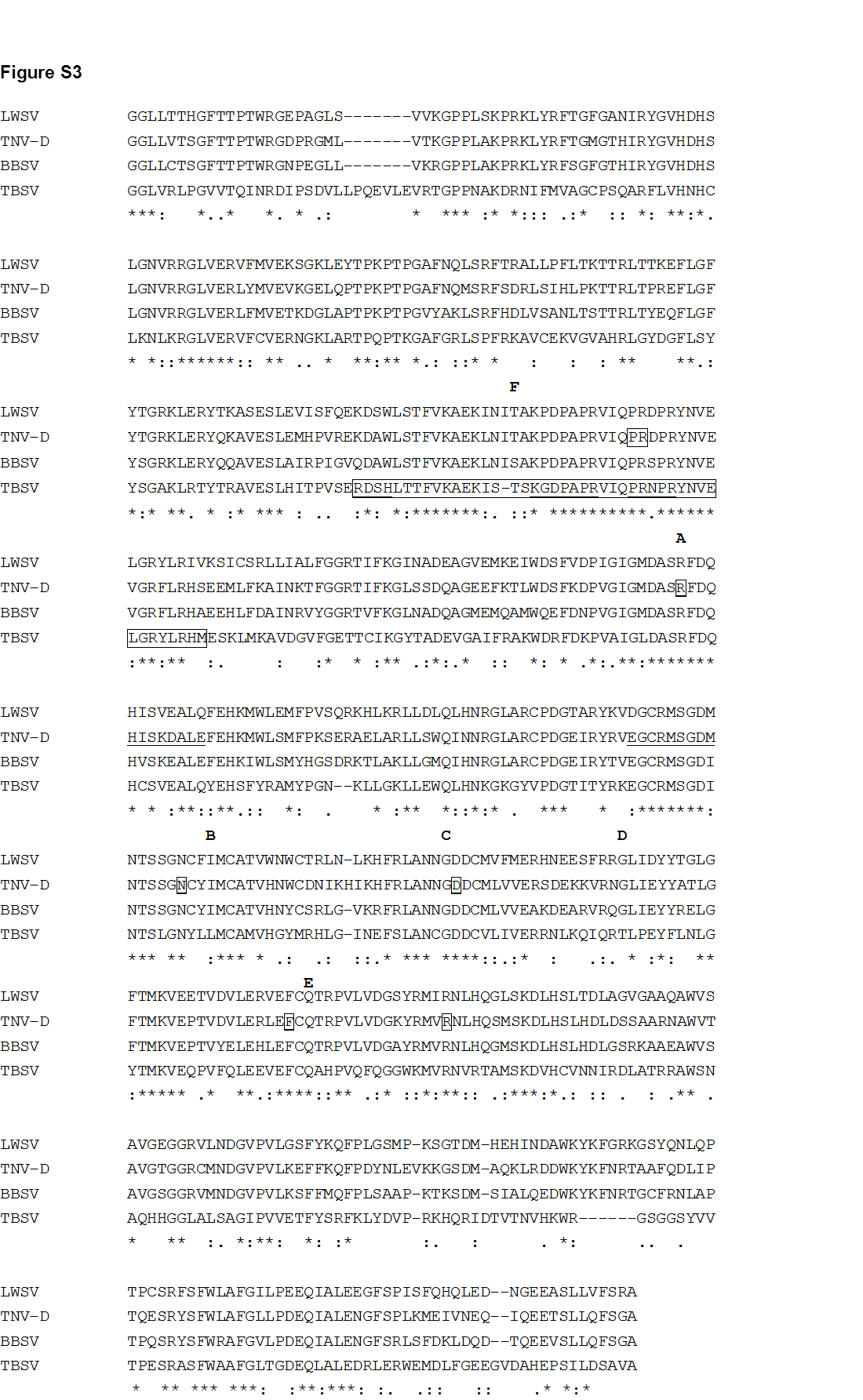

Supplement: Figure S3 — Amino acid alignment of the putative polymerase domains from members of the genus Betanecrovirus and TBSV which are most closely related to TNV-D. Accession numbers of each virus are shown in brackets: Leek white stripe virus (LWSV; NC001822) TNV-D (D00942), Beet black scorch virus (BBSV; FN565520) and TBSV (M21958). The amino acid sequences depicted show the readthrough domain of the putative RdRPs immediately downstream of the amber termination codon and extend to the termination codon of the ORFs. The sequences were aligned using the Fast Fourier Transform MAFFT program L9INS-1 [24]. The conserved motifs (A-E) within the domains [10] are underlined in the TNV-D sequence and the amino acids mutated in this investigation are boxed. The RNA binding motif, RBR2 (F motif) is boxed in the TBSV sequence and the RdRP palm sub-domains [13] are underlined. Asterisks signify identical residues; colons signify highly conserved amino acid residues; single dots less conserved, but related residues in the four sequences. (TIF) [file pone.0080912.s003.tif]
